# Smart Prognostics and Health Management (SPHM) in Smart Manufacturing: An Interoperable Framework

**DOI:** 10.3390/s21185994

**Published:** 2021-09-07

**Authors:** Sarvesh Sundaram, Abe Zeid

**Affiliations:** College of Engineering, Northeastern University, Boston, MA 02135, USA; sundaram.s@northeastern.edu

**Keywords:** Smart Manufacturing, Smart Prognostics and Health Management, data preparation, interoperability, Data Mining, Machine Learning, Deep Learning

## Abstract

Advances in the manufacturing industry have led to modern approaches such as Industry 4.0, Cyber-Physical Systems, Smart Manufacturing (SM) and Digital Twins. The traditional manufacturing architecture that consisted of hierarchical layers has evolved into a hierarchy-free network in which all the areas of a manufacturing enterprise are interconnected. The field devices on the shop floor generate large amounts of data that can be useful for maintenance planning. Prognostics and Health Management (PHM) approaches use this data and help us in fault detection and Remaining Useful Life (RUL) estimation. Although there is a significant amount of research primarily focused on tool wear prediction and Condition-Based Monitoring (CBM), there is not much importance given to the multiple facets of PHM. This paper conducts a review of PHM approaches, the current research trends and proposes a three-phased interoperable framework to implement Smart Prognostics and Health Management (SPHM). The uniqueness of SPHM lies in its framework, which makes it applicable to any manufacturing operation across the industry. The framework consists of three phases: Phase 1 consists of the shopfloor setup and data acquisition steps, Phase 2 describes steps to prepare and analyze the data and Phase 3 consists of modeling, predictions and deployment. The first two phases of SPHM are addressed in detail and an overview is provided for the third phase, which is a part of ongoing research. As a use-case, the first two phases of the SPHM framework are applied to data from a milling machine operation.

## 1. Introduction

The modern manufacturing era has enabled the collection of large amounts of data from factories and production plants. Data from all levels of an enterprise can be analyzed using Machine Learning (ML) and Deep Learning (DL) techniques. Interdisciplinary approaches such as Industry 4.0 (I4.0), Cyber-Physical Systems (CPS), Cloud-Based Manufacturing (CBM) and Smart Manufacturing (SM) allow the real-time monitoring of operations in manufacturing facilities. These approaches greatly benefit maintenance operations by reducing downtime and thereby cutting costs. Monte-Carlo estimations suggest that annual costs concerning maintenance amount to approximately USD 222 Billion in the United States [1], and recalls due to faulty goods result in costs of more than USD 7 Billion each year [2]. One of the reasons for these relatively high costs is manufacturing organizations preferring corrective or preventive maintenance as opposed to predictive maintenance. Prognostics and Health Management (PHM) is an interdisciplinary area of engineering that deals with the monitoring of system health, detecting failures, diagnosing the cause of failures and making a prognosis of component and system level failures by using metrics such as Remaining Useful Life (RUL). PHM technologies are being widely incorporated into the modern manufacturing approaches as an in situ evaluation of the system is made possible.

**Figure 1:**
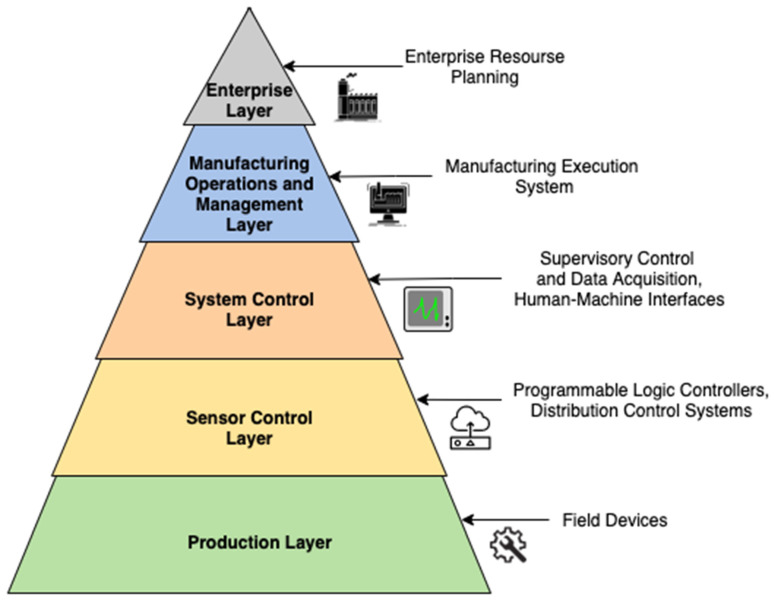
Top-down approach to manufacturing system according to ISA-95 Automation Pyramid.

A Smart Manufacturing (SM) paradigm consists of interoperable layers that are capable of vertical as well as horizontal integration [3]. Figure 1 shows the different layers according to the ISA-95 Automation Pyramid. The physical layer of SM consists of the field devices that comprise of sensors and actuation equipment. To monitor and analyze the devices on the physical layer, data from all the individual components need to be incorporated into a single stream, which then provides us with a context of the entire operation. Not only do the data give us context about the operation, but properly formatted data allow faster deployment of AI and ML algorithms. Quicker implementation of ML- and DL-based condition monitoring techniques allows early detection of potential failures. The data generated come from various sources involving multiple parameters, resulting in complex formats and sometimes redundancies. Challenges in effective implementation of PHM techniques for predictive maintenance include the availability of data, the preparation of data and ensuring that appropriate ML and DL methods are selected for modeling. Appropriate steps need to be taken to ensure that manufacturing data can be acquired and preprocessed for PHM.

To address these requirements, this paper proposes a Smart Prognostics and Health Management (SPHM) framework in SM. To that end, the rest of the paper is structured as follows: Section 2 provides a review of existing approaches to maintenance and state-of-the-art PHM methods. Section 3 identifies the research gap based on existing work. Section 4 proposes an interoperable framework to implement SPHM in SM. Section 5 applies the first two phases of the proposed framework to data obtained from a milling operation. Section 6 discusses the results. Finally, Section 7 provides the conclusion and future work.

## 2. Review of Key Concepts and Trends

### 2.1. Overview of Maintenance Strategies

In recent years, maintenance engineering has emerged as one of the most important areas in manufacturing organizational planning as opposed to it being purely associated with production operations [4]. Proactive approaches to maintenance are being adopted by manufacturers, not only as a cost-cutting measure, but also as a competitive strategy [5]. Manufacturing enterprises implement maintenance strategies based on production requirements, the complexity of machinery and equipment, and the costs involved. Broadly speaking, maintenance strategies follow one of the three main approaches:

- Unplanned or reactive maintenance—typically allows for machinery to breakdown, after which it is analyzed and repaired.
- Planned or preventive maintenance—an assessment of the system is conducted at regular time intervals to determine whether any repair/replacement is necessary. It is important to note that the health of the system is not taken into consideration in establishing the time intervals.
- Predictive maintenance—a data-driven approach in which parameters concerning the health of the system are used to monitor the condition of the equipment and in determining the RUL.

Approaches to maintenance strategies have evolved over the years with the advent of data analytics and advanced ML/DL techniques. Continuous monitoring of shop floor operations with the use of Internet of Things (IoT), smart sensors and smart devices are allowing organizations to quickly make cost saving-decisions, as opposed to a long and drawn-out analysis that has proven to be very costly. Enterprises are evolving their maintenance strategies based on data-driven approaches and the value of predictive maintenance is being realized from its results.

### 2.2. Multi-Faceted Approach to PHM

Condition-Based Monitoring (CBM) techniques and Remaining Useful Life prediction are important features of predictive maintenance strategies leading to PHM methods. It would be remiss to state that CBM and RUL define PHM methodologies, since PHM methods are multi-faceted approaches. PHM methods consist of: data acquisition and preprocessing, degradation detection, diagnostics, prognostics and timely development of maintenance policies for decision making [6,7]. A brief overview of PHM’s many facets is provided as follows:

1. Data acquisition and preprocessing: For any predictive problem in maintenance to be solved, the availability of data is of utmost importance. IoT devices and smart sensors are typically used to acquire data in manufacturing settings. The data are recorded and evaluated in real-time as certain anomalies may be detected at an early stage by maintenance engineers or control systems. The collection of such data is extremely important as it provides vital information that helps to understand the relationships between the heterogenous components of the system. Once the data are collected, they are analyzed and preprocessed to ensure that crucial information which helps in failure detection is obtained.
2. Degradation detection: Identifying that a component is degrading or that it is bound to fail is the next step once the data have been collected and prepared. Anomalies and failures can be detected using sensor readings and by other specified criteria, such as surface roughness, temperature, size of tools/equipment, etc.
3. Diagnostics: Once a determination is made that a failure is occurring, understanding the cause of the failure is the next step. Failure types can be categorized to evaluate the extent of the failure, helping in finding its root causes. Operating conditions of individual components can be analyzed along with their interactions to help diagnose the cause of failures.
4. Prognostics: With the ability to detect failures using diagnosing mechanisms, predictive methods are used to predict the system health to avoid potential failures. Model-based prognostics involve Physics-of-Failure (PoF) methods to assess wear and predict failure. However, such approaches are limited as even minor changes to the operations can result in poor predictive power. Data-driven approaches are becoming more common for prognostics with the use of DL and ML techniques. By using data-driven methods along with crucial information from physics-based methods, highly accurate predictions can be made about systems.
5. Maintenance decisions: Based on results from the predictive methods developed, manufacturing enterprises can determine policies to be followed for maintenance planning that will help with less downtime, higher yield and a reduction in losses.

The importance of enumerating the many phases of PHM is to help us understand that predictive maintenance is an aggregation of methods from data engineering, reliability and quality engineering, material sciences, DL, ML and organizational decision making. While PHM methods in manufacturing face several challenges, a fundamental one is a requirement for an interoperable approach that allows its implementation across different industries.

### 2.3. Challenges in Implementing PHM in the Industry

While there is significant research being conducted on the different areas of PHM, there are some challenges as well. Researchers from the National Institute of Standards and Technology (NIST) outline some of the most significant challenges in PHM that can be categorized as follows [8].

#### 2.3.1. In Prognostics

- Insufficient failure data or excessive failure data may skew prediction of RUL
- Inadequate standards to assess prognostic models
- Lack of precise real-time assessment of RUL
- Uncertainty in determining accuracy and performance of prognostic models.

#### 2.3.2. In Diagnostics

- Expertise required in diagnosis of failures
- Limitations due to lack of training and formal guidelines in authentication of diagnostic methods
- Difficulty in diagnosis due to outliers, noise in signal data and operating environment.

#### 2.3.3. In Manufacturing

- Ability to effectively assess electronic components
- Integration of sensors and field devices with PHM standards
- Inconsistencies in data, data formats, and interoperability of data in manufacturing facilities
- Inadequate correspondence between production planning and control units and maintenance departments
- High level of complexity and heterogeneity in manufacturing systems.

#### 2.3.4. In Enterprises

- Proactive involvement required towards maintenance to view PHM as a cost-saving approach and not a cost-inducing one
- Enterprises with legacy machines and equipment tend to go with one of the traditional approaches to maintenance, even though PHM methods are more effective
- Securing funding for PHM projects.

#### 2.3.5. In Human Factors

- User friendly interfaces and applications
- Collection of expert knowledge
- Improvement in outlook towards implementing changes to existing mechanisms.

Most of the research being conducted is aimed at monitoring system health and RUL in PHM, while the other areas of PHM are not given as much importance. Our aim is to address some of the difficulties faced throughout all the phases of PHM. Since prognostics is one of the most significant areas in PHM, we look at some of the modeling approaches.

### 2.4. Overview of Prognostics Modeling Approaches

There are three main prognostics approaches to PHM modeling: physics-based models, data-driven models and hybrid models that are a combination of physics-based and data-driven models [9]. While all three approaches are used in industry, the application of prognostics modeling also faces several challenges [10]:

- Lack of readily available data in a standardized format
- Insufficient failure data due to imbalance in data classes
- Lack of physics-based parameters in the data.

Industrial data from manufacturing systems are also often complex and require a great deal of preparation to be acceptable for modeling. Due to these reasons, efforts are required to prepare and preprocess the data to make them suitable for prognostics modeling. Data-driven and hybrid models are often preferred over physics-based models given the flexibility of analytical techniques that can be used. A synopsis of physics-based, data-driven and hybrid models based on [9,10,11,12] is outlined in Table 1.

In the last two decades, there have been great strides made in improving physics-based prognostic approaches. Several of these approaches are reviewed and applied to rotating machinery by Cubillo et al. [13]. PoF methods have been tested on electronic components in monitoring the health of electronic components by Pecht et al. [14]. The RUL of lithium-ion batteries has been predicted by physics-based models in [15]. There are also several publications that address prognostics modeling based on evolutionary methods derived from bio-inspired [16,17] and neuro-inspired algorithms [18,19]. However, based on current research and industry trends, we will limit our focus to data-driven approaches.

### 2.5. Current Trends in PHM Research

Advanced algorithms and optimization techniques are at the forefront of problem solving in PHM areas, and a review of the current state of research is necessary to understand these topics. ML and DL have become the choice of modeling techniques in studies that undertake data-driven approaches. While there is an abundance of analytical techniques available, there are few publicly available datasets for PHM research. Most datasets are limited to those released by academic institutions and government organizations. This has resulted in certain datasets being benchmarked to test prognostics models, as seen in [10,20]. Datasets that have been used in PHM research are often from PHM data challenges, and the modeling objective can be grouped into four main tasks: prognosis, fault diagnosis, fault detection and health assessment. An in-depth discussion of these datasets can be found in the review conducted by Jia et al. [21]. Another area from which PHM can be approached is from the perspective of manufacturing models. These manufacturing approaches are designed keeping in mind the objectives of maintenance and PHM policies. We will now shift our focus to ML, DL, Health Index and manufacturing approaches that are the frontiers of PHM research.

#### 2.5.1. Applications of Machine Learning in PHM

ML models have applications in a wide range of PHM areas. Although most of the research is focused on CBM and RUL prediction, some papers focus on fault detection as well. An extensive study on the use of Support Vector Machines (SVM) in RUL prediction was conducted by Huang et al. [22]. The authors investigate how SVM works in condition monitoring in a real-time setting, as well as in future RUL predictions. Mathew et al. [23] propose several supervised ML algorithms, such as Decision Trees, Random Forest, k-Nearest Neighbors (kNN) and regression, in estimating the remaining lifecycles of aircraft turbofan engines by a comparison of the Root Mean Square Error (RMSE) metric. It was identified that the random forest model performed best in this setting. Researchers in [24] compare the performance of neural networks, Support Vector Regression (SVR) and Gaussian regression on data from slow-speed bearings that consist of acoustic emission readings. Implementations of techniques such as Least Absolute Shrinkage and Selection Operator (LASSO) Regression, Multi-Layer Perceptron (MLP), SVR and Gradient-Boosted Trees (GBT) are tested on data collected from Unmanned Aerial Vehicles (UAV) in [25]. In this case, non-linear techniques were preferred over linear models, with the best performance achieved by GBT. In fault detection, an SVR outperforms multiple regression on a milling machine dataset, especially when more data are used from sensors [26]. A review of Machine Learning techniques used in intelligent fault detection was conducted by Lei et al. [27], and their challenges were outlined. It is important to note that there are several studies that use semi-supervised ML methods in fault detection of manufacturing equipment, as seen in [28,29,30], but a discussion of these topics is beyond the scope of this paper.

#### 2.5.2. Applications of Deep Learning in PHM

Deep Learning (DL) methods have evolved as frontrunners in RUL assessment largely due to the deep architectures deployed and the ability to tweak the optimization parameters. Recurrent Neural Networks (RNNs) are popular DL methods used in PHM due to their wide range of applicability. Malhi et al. [31] focus on preprocessing of signals using wavelet transformation and apply RNN to investigate its effects on performance. Heimes [32] uses RNN with an Extended Kalman Filter (EKD), backpropagation and Differential Evolution (DE). Research conducted by Palau et al. [33] implemented a Weibull Time-To-Event (WTTE) method with an RNN to predict time-to-failure and demonstrate how it affects real-time distributed collaborative prognostics. A novel method using embedded time series measurements that does not take into consideration any prior knowledge about machine degradation was developed by Gugulothu et al. [34]. Recently, probabilistic generative modeling using Deep Belief Networks (DBN) are being used for feature extraction and in RUL estimation. Authors in [35] argue that feature extraction from data belonging to SM and I4.0 manufacturing can be troublesome due to requirements of extensive prior knowledge, and deploy a Restricted Boltzmann Machine (RBN)-based DBN to estimate RUL. Another interesting study by Zhao et al. [36] uses DBN to extract features, supplemented by a Relevance Vector Machine (RVM) in the prediction of RUL of battery systems. A multi-objective DBN ensemble using evolutionary algorithms was employed in RUL prediction of turbofan engines by [37]. Methods such as RBM have also been implemented with regularization to generate features that are correlated with fault detection criteria [38]. Convolutional Neural Networks (CNNs) have also been used for machine health monitoring with one-dimensional data in [39,40,41,42] and for feature extraction and automated feature learning with two-dimensional data in [43,44,45,46,47,48]. Comprehensive reviews of Deep Learning methods in PHM, such as Autoencoders, RNNs, RBM and DBN, were conducted by Khan et al. [49].

#### 2.5.3. Health Index Construction

Another important area in health monitoring and management is the construction of a Health Index (HI) from input data parameters and using the HI in fault detection and prognostics. HI’s are developed using Principal Component Analysis (PCA), and similarity matching by using distance measures for RUL estimation of a factory slotter is analyzed by Liu et al. [50]. In lifecycle prediction of battery systems, Liu et al. [51] develop a novel technique to extract HI while preserving important degradation information. RUL predictions using HI compared to ones without explicit HI on data from induction motors show that HI-based RUL prediction is preferred [52].

#### 2.5.4. PHM Using Manufacturing Paradigms

Over the last few decades, manufacturing environments have been revolutionized from a multi-objective viewpoint. Not only are costs and yield the sole objectives, but product customization, sustainability, modularity in the shopfloor and servitization are equally important. This multi-objective approach to manufacturing has allowed the area of PHM to be built-in while designing new systems. Approaches such as mass customization, reconfigurable manufacturing, service-oriented manufacturing and sustainable manufacturing allow the incorporation of PHM goals within the manufacturing system’s setup [53].

Mass customization is a multi-dimensional approach to manufacturing that deals with product design, manufacturing processes and the manufacturing supply chain [54]. This approach presents a shift in the traditional manufacturing objective, changing it from a high production volume with a low variation in products to a high variety of products with a lower production volume. This of course presents its own challenges to PHM due to the sheer number of customizations required in production processes. To tackle this, maintenance policies are integrated based on condition monitoring of systems and order volumes by Jin and Ni [55]. Decisions can also be made from a cost-based perspective by including all the maintenance and production costs in the objective [56].

Reconfigurable manufacturing systems (RMS) are modular in their design, allowing changes in their structure to adjust to any inherent changes or shifts in market demands [57]. RMS systems have maintenance policies dependent on their structure: parallel, series, series-parallel, etc. [58]. Preventive maintenance-based RMS was developed by Zhou et al. [59]. The objectives of reduce, reuse, recycle, recover, redesign and remanufacturing were incorporated into RMS to improve the response time to manage system health by Koren et al. [60].

Service-oriented manufacturing offers a Product Service System (PSS), allowing products and services to be picked based on customer needs [61]. PHM services can be offered depending on the manufacturer’s needs, enabling a highly customized approach to PHM services. To maximize the prognostic and diagnostic capabilities of Original Equipment Manufacturer (OEM), a cloud-based approach has been developed by Ning et al. [62].

## 3. Research Gap and Proposal

The focus of many studies has been the implementation of ML and DL algorithms in identifying RUL of machinery and equipment. While most publications aim to test the predictive power of data-driven models, very few enumerate all the steps taken required to implement PHM methods in manufacturing. Traini et al. [63] developed a framework to address predictive maintenance in milling based on a generalized methodology. Yaguo et al. reviewed the stages in CBM, from data acquisition to RUL estimation, for different PHM datasets. Mohanraj et al. [64] reviewed the steps in condition monitoring from the perspective of a milling operation. A framework for PHM in manufacturing with cost–benefit analysis was developed by Shin et al. [65], and a use-case for data on batteries was evaluated. While these publications provide noteworthy steps to implement prognostics models in the industry, there are no frameworks to address modern PHM approaches to SM, with an in-depth understanding of all the steps involved. To address this research requirement, we introduce Smart Prognostics and Health Management (SPHM) and propose an interoperable framework for SPHM in SM.

## 4. An Interoperable Framework for SPHM in SM

**Figure 2:**
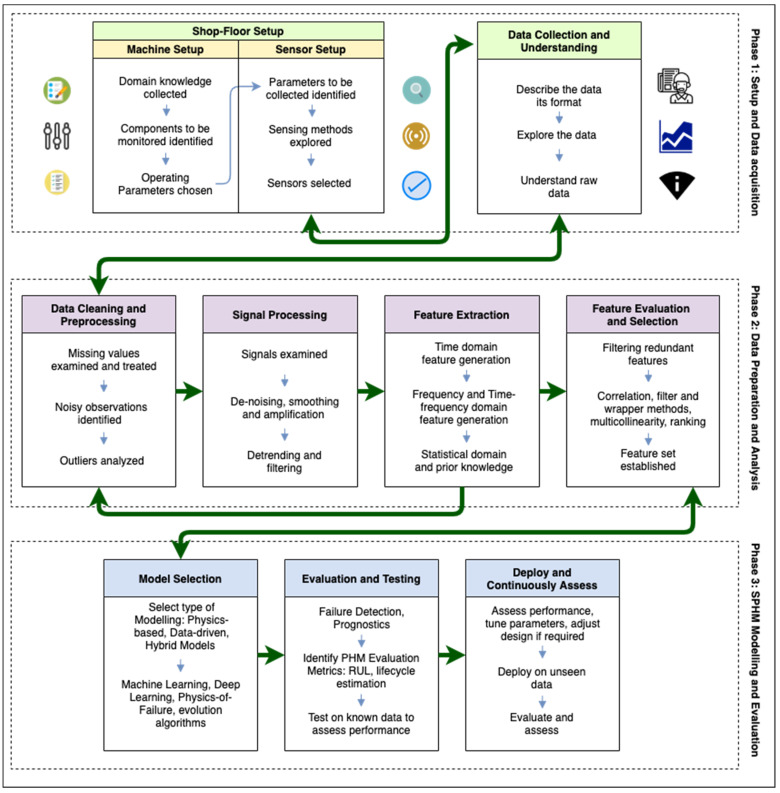
An interoperable framework for Smart Prognostics and Health Management (SPHM) in Smart Manufacturing (SM).

Current approaches to PHM are tailor-made to individual systems and components on the shopfloor. Predictive maintenance methodologies, although applicable to heterogeneous machinery, are generic in their approach, without specifying the particulars of data acquisition, modeling and applications. SPHM is a concept that addresses the many facets of PHM in Smart Manufacturing in an interoperable manner. SPHM’s uniqueness lies in its interoperable framework, that addresses all the specifics of PHM by using Industry 4.0 and SM standards in a phased approach. The structure of the framework is loosely based on The Cross-Industry Standard Process for Data Mining (CRISP-DM) [66], an industry standard to apply DM and ML keeping business objectives in mind. The framework consists of three interconnected phases, each with the objective of addressing some of the challenges discussed previously. The proposed SPHM framework for SM with all its phases is shown in Figure 2. The setup and acquisition of data from the shopfloor are covered in the first phase, the preparation of the data and an analysis of the various parameters collected in the data, including an understanding of signal processing, are enumerated in the second phase, and the modeling approaches to SPHM along with their evaluation are discussed in the third phase.

This paper provides an in-depth description of the first two phases of the SPHM framework, and an overview of the methods in the third phase. A detailed discussion and implementation of modeling techniques are part of ongoing research.

### 4.1. Phase 1: Setup and Data Acquisition Phase

#### 4.1.1. Shopfloor Setup

The first phase in the framework involves identifying the machinery or equipment that are going to be assessed, using knowledge from the maintenance and production departments. Prior domain knowledge from engineers and technicians will help us identify which components are crucial to the operation. In manufacturing operations, these components are often tool tips or bearings. Once the identification of components has been established, the next step would be to collect information about the operating parameters and environmental conditions. It is important to note that interacting factors also need to be considered in this step. A detailed report with all data about the operating parameters is prepared and a discussion is held with engineers about the relevance of the parameters. Setting up the equipment and sensors is the next part of this step. Most machinery already come with preinstalled sensors, for example Computer Numeric Control (CNC) machines often consist of sensors to capture electric current, vibration, acoustic emission, spindle, torque, etc. The information from these sensors is vital in analyzing the health of the machine. If additional sensors are required, or if the identified equipment consist of legacy machinery, sensors will have to be retrofitted. Often, there may be sensors that are installed for human-factor purposes, essentially aimed at operator safety. These sensors may have no effect on any prognostics or fault detection methodology, so they may be ignored for selection based on existing knowledge. Using information from operating parameters and domain knowledge, a detailed step-by-step guide to setup and run the machine is produced. Once a strategy is in place to conduct the experiment under standard operating conditions, the next step is to identify an appropriate data collection methodology.

#### 4.1.2. Data Collection and Understanding

One of the first and arguably most important considerations in this step is to identify how and where to store the data from operations and devices. A requirement in most organizations is the ability to access data in a secure manner. A key aspect of interoperability is the ability to access data by tasks that share it [3]. Cloud-based systems and Big Data platforms provide secure access to data by using cybersecurity mechanisms, ensuring no misuse of the data. Systems can be setup to directly upload and download data that have been collected from the shopfloor. The collected data need to be analyzed and thoroughly reviewed to ensure there were no inconsistencies encountered during the acquisition of data. The data files should also be recorded in a format such that is readable and its suitable for information extraction. Preliminary investigation of the data by using exploratory analyses such as signal plots, range of attribute values and a basic statistical review will help to gain a better understanding of the experiment. Information such as sampling rate and frequencies pertaining to signal measurements should be recorded. Any other a priori information that would aid in describing the data should also be included in this step.

### 4.2. Phase 2: Data Preparation and Analysis

In the data preparation phase, there are a few key steps that need to be followed: data cleaning and preprocessing, signal preprocessing, feature extraction and feature evaluation and selection. These steps are paramount to successful implementation of CBM and predictive maintenance methods as they preserve and extract information from the data that best represents the physical experiment.

#### 4.2.1. Data Cleaning and Preprocessing

The procedure to clean and preprocess the data can be understood best by posing the following four questions: (1) Are there any missing values? (2) Is there any noise in the data? (3) Are there any outliers or skewed measurements? (4) Are the data on the same scale? Data cleaning and preprocessing involve many more techniques depending on the type of data recorded, but the treatment of missing values, noise, outliers or skewed instances and scaling and normalization are some of the most crucial ones to be considered.

Missing values can be problematic in any data. Large numbers of missing values can affect the analysis by introducing a bias, causing skewness. Missing value treatment generally involves either the deletion of the record if permissible or replacement of the missing value by using imputation methods [67,68]. Commonly used imputation techniques are mean, median or mode imputation. In this case, the missing values are replaced by the respective mean, median or mode of all the values of that attribute. Predictive modeling methods such as regression and kNN are also used for imputation. However, it is critical to understand which attributes contain missing values. If it is the dependent variable, the strategy most likely to be used would be the removal of the instances that contain the missing values, since we have no control over the values in that attribute. In the case of independent variables, we may have the freedom to use one of the imputation methods discussed previously.

Noise in the data is generally caused due to errors in measurement, causing corrupted data. This may cause inconsistencies in modeling and analysis, and possibly provide incorrect results. Examples of noise are duplicate records and mismatch in data types. The ideal method to address noise is to remove those instances affected. If there is significant noise in the attributes, those values may be removed individually, and replaced using one of the imputation methods. Noise in the data may also be caused due to probabilistic randomness. It is important to note that this type of noise, also known as random errors, can be very difficult to predict. These values will have to be examined and are generally retained as they can be accounted for as randomness in measurements. It is essential to note that the noise we discuss here is different from the noise in signals. We will discuss noise in signal readings in the signal preprocessing step.

To answer the third question, outliers can be due to a variety of reasons. Outliers may be genuine observations that exist for a reason. For example, the readings from the data the moment a tool breaks can be considered as outliers. The values of the readings from that instance may be outliers compared to other readings, but they are still genuine readings since they convey essential information. Outliers in the data may also be due to systematic errors or measurement errors. These outliers are problematic since they can be attributed to flaws in readings and convey no genuine information. The preferred solution to address these outliers is by simply removing them from the data. There may also be differences in measurement criteria that may cause inherent skewness and will have to be treated. Sometimes, attributes in the existing format may not convey the information that is necessary due to some form of skewness. The skewness does not necessarily mean there is something wrong with the data. Instead, it could be due to a small sample size or due to intrinsic factors. In such cases, a transformation may be necessary. Commonly used transformations are log, power, square root, etc. It may also be deemed necessary to use a transformation when the attributes are non-linear and need to be linear to form a meaningful relationship with the other attributes or the target.

Another consideration to be made in this step is the process of scaling or normalizing the data. Normalization or standardization may be required if the data recorded are on varying scales. Having feature values on different scales can cause some features to have more strength in predictions, which is often erroneous. Following are some commonly used methods to scale and normalize data:

1. Min–max normalization

Normalizing data using min–max is a method that shifts all the data values to a scale between 0 and 1. The minimum and maximum values are recorded, and Equation (1) is applied to scale it.

(1) $$x^{\prime }=\frac{x-\min\left(x\right)}{\max\left(x\right)-\min\left(x\right)}$$

2. Mean normalization

Mean normalization is another method that centers the data around the mean, as shown in Equation (2):

(2) $$x^{\prime }=\frac{x-\mathrm{mean}\left(x\right)}{\max\left(x\right)-\min\left(x\right)}$$

3. Unit Scaling

For vectors that consist of continuous values, scaling them to a unit length maintains the same direction, but changes its magnitude to 1. See Equation (3):

(3) $$x^{\prime }=\frac{x}{\left|\left|x\right|\right|}$$

4. Standardization

Standardization involves making a loose distributional assumption about the data and scaling it around the mean. Z-Score standardization involves making a normal distributional assumption and using the mean value (μ) and standard deviation (σ), as seen in Equation (4):

(4) $$x^{\prime }=\frac{x-\mu }{\sigma }$$

The steps involved in preprocessing are applied to new features that are extracted/generated as well. Hence, it is required to note that in the data preparation and analysis phase, the steps go back and forth.

#### 4.2.2. Signal Preprocessing

The data collected from sensor measurements in most cases are in the form of signals. Signal processing is a complex field of study and involves identifying the type of signal and modifying it in some form to improve its quality. There are a few actions that need to be taken to enhance the quality of signals. First, we need to consider the type of signals that exist in the data. In manufacturing data, we usually observe signals from sound, vibration and power. Since these signals are observed across different machines in the industry, we will focus our discussion on preprocessing these signals. Signal preprocessing involves some key tasks, such as denoising, amplification and filtering. We will discuss these topics in brief in this section.

Noise reduction methods or de-noising is a process that diminishes noise in the signals. The entire removal of noise is not possible, so curtailing it to an acceptable limit is the goal of this step. The Signal-to-Noise Ratio (SNR) is a metric used to determine how much of the signal is composed of true signal versus noise. Signals are decomposed using techniques such as wavelet transforms and median filters [69], which preserve the original signal while reducing the amount of noise that it is composed of. Signal amplification is also a method used in signal processing that improves the quality of the signal by using one of two approaches: boosting its resolution or reducing SNR. Signals are generally amplified to meet threshold requirements of equipment being used.

Filtering is one of the steps in signal conditioning, in which low-pass and high-pass filters are used in attenuating signals based on a specified cut-off. Low-pass filters block high frequencies while allowing low frequencies to pass, and high-pass filters block low frequencies while allowing high frequencies to pass through. Low-pass filters help in removing noise, and high-pass filters filter out the unwanted portions and fluctuations of signals. The cut-off frequencies are generally chosen depending on the noise observed.

There are also methods to pre-process signals using statistical techniques. Estimation methods such as Minimum Variance Unbiased Estimator (MVUE), Cramer-Rao lower bound method, Maximum Likelihood estimation (MLE), Least Squares Estimation (LSE), Monte-Carlo method, method of Moments and Bayesian estimation, along with several others, are discussed in the context of signal processing in [70]. We will not further discuss these estimation methods in signal processing as it is beyond the scope of this article.

#### 4.2.3. Feature Extraction

Signal measurements recorded are high in dimensionality and consist of readings that cannot be directly used in any form of modeling. This is due to the non-linearity of the machine operation that is also dependent on time [71]. To understand the signal readings in the context of the manufacturing process in question, the relevant information from the signals should be extracted or generated. This information from the signals is extracted as features for the dataset. These features are extracted in the time domain, frequency domain and time-frequency domain. Zhang et al. [72] have compiled a list of features that can be extracted in all three of these domains for machining processes. An overview of these features is as follows.

##### Time-Domain Feature Extraction

There are several statistical features that can be extracted from signals in the time domain. Features such as maximum value, mean value, root mean square, variance and standard deviation are extracted. Additionally, higher-order statistical features such as kurtosis and skewness are calculated. These values are dependent on the probability distribution function, with kurtosis providing information about the peak of the distribution and skewness explaining if the distribution is symmetrical or not. The Peak-to-Peak feature computes the difference between extreme values of the amplitude, i.e., difference between maximum and minimum values. Crest factor is the ratio of the maximum value and mean values of the signal. A list of time-domain features and their description is provided in Table 2. We refer readers to [72,73] for in-depth explanations of the features.

##### Frequency-Domain Feature Extraction

Features in the frequency domain are obtained by using a transform on the signal signatures. The Discrete Fourier Transform (DFT) is a method used in spectral analysis of signals. The DFT is based on the Fourier Transform method, see Equation (5) [74]:

(5) $$X\left(\omega_{x}\right)=\sum_{n=0}^{N-1}x\left(t_{n}\right)e^{-jw_{k}t_{n\;}}\;\mathrm{for}\;k=0,\;1,\;2,\;\ldots ,\;N-1\;$$

where,

$x\left(t_{n}\right)$ = input signal at time $t_{n}$,
$t_{n}$ = *nT =* n-th sampling instant, for n ≥ 0,
$X\left(\omega_{x}\right)$ = spectrum of *x* at frequency $\omega_{k}$,
$\omega_{k}$ = sample from k-th frequency in radians per second,
*T =* sampling interval in seconds,
$f_{s}$ = 1/*T* = sampling rate or samples per second,
N = total number of samples in signal.

The Discrete Time Fourier Transform (DTFT) is a limiting form of the DFT allowing infinite samples, see Equation (6):

(6) $$X\left(\omega^{*}\right)=\sum_{n=-\infty }^{\infty }x\left(n\right)e^{-j\omega n}$$

where,

x(n) = signal amplitude at $n^{th}$ sample,
$X\left(\omega^{*}\right)$ = DTFT of *x* at $n^{th}$ sample.

The Fast Fourier Transform (FFT) is an algorithm that uses the Discrete Fourier Transform (DTF) method to convert the signal measurements from the original order to the frequency domain by sampling them over time, which is most used for practical purposes. The number of samples is an important parameter to be noted in this stage and is used in the calculation of power spectral density of the signals using a periodogram [75], which is nothing but a ratio of the squared magnitude of DTTF and the number of samples, see Equation (7):

(7) $$P_{x,\;M\left(\omega \right)}=\frac{{\left|DTTF\right|}^{2}}{N}$$

For a more comprehensive understanding of Fourier Transforms, DFT, DTFT and periodograms, refer to Smith [74,75]. Features such as maximum, sum, mean, variance, skewness, kurtosis and relative spectral peaks extracted from power spectra are commonly extracted from signals that are produced by machining operations. The list of frequency-domain features is shown in Table 3. Zhang et al. [72] and Caesarendra et al. [73] provide more details for these frequency-domain features.

##### Time-Frequency Domain Features

Like frequency-domain feature extraction, time-frequency features are extracted by using wavelet transforms. Time-frequency analysis provides relationships represented both over time and frequency. This two-dimensional view of the signal, in some cases, can generate features that are not captured by time-domain features or frequency-domain features. Methods such as Short-Term Fourier Transform (STFT), Continuous Wavelet Transform (CWT), Wavelet Packet Transform (WPT) and Hilbert-Huang Transform (HHT) are used to extract features in the time-frequency domain [72,76]. These methods analyze the signals on a two-dimensional view by using a combined function for the two domains. Although there is much research conducted in this field, an elaboration on these methods is beyond the scope of this paper.

#### 4.2.4. Feature Evaluation and Selection

The feature extraction or feature generation from signals results in a high-dimensional space with many features. There are often more features than the number of recorded instances after the feature extraction step. This could be troublesome, causing any modeling technique to potentially overfit the data, causing misleading results. Overfitting is caused by some form of redundancy in the high-dimensional space, with not all features being related to the predictor or dependent variable. A solution to this problem is reducing the number of features that are used in modeling. Feature evaluation and selection methods select features that are better in predicting the response variable than all the other features in the feature space. They primarily fall into three main categories: filter methods, wrapper methods and embedded methods.

Filter methods calculate the performance of features across the entire dataset and select the top-performing features. The most used filter method for feature selection is correlation analysis. There are different computational approaches to calculating the correlation coefficient, and Pearson’s, Kendall’s and Spearman’s correlation are the ones that are commonly used. In correlation analysis, pairwise correlation coefficients are calculated between the variables and compared. The analysis is aimed at eliminating features from highly correlated pairs. In a highly correlated pair, either one of the features can be used in prediction while the other is eliminated. There is a concern with this approach, however, as not all features in the dataset may have a relationship with the dependent variable. This may result in a useful feature being dropped during the correlation analysis. To circumvent this issue, one may sometimes calculate the correlation of all the features in the data against the response and use this to eliminate the weaker feature in the pair. Correlation analyses are effective in feature selection. Cross-validation, a method in which data are divided to validate any analysis or modeling, is sometimes used with correlation for feature selection. Methods such as Random Forest to calculate feature importance, Mutual Information to obtain the entropy, analysis of variance (ANOVA) and several others are compared in [77].

Wrapper methods use subsets of the features to identify which ones are more important to the dataset. These methods generally deploy search-based algorithms to find the best features from the feature space. Wrapper methods can be broadly classified into two categories: heuristic search methods and sequential search algorithms [78]. Heuristic methods include Genetic algorithm (GA), Variable Neighborhood Search (VNS), Simulated Annealing (SA), Particle Swarm Optimization (PSO), etc. Sequential search algorithms include Forward Selection, a method in which an empty feature set is used, and features are added individually and evaluated using modeling techniques. Backward Selection is another sequential search method in which the entire feature set is used, and features are evaluated and eliminated depending on model performance. In an exhaustive search method, subsets of features are evaluated against model performance and the best subset is chosen.

Embedded feature selection methods are deployed on subsets of the data along with the modeling techniques. Regularization is an important method in which a penalty is added to the model as a constraint. The regularizer penalizes the coefficients of features in a model, thereby reducing the feature’s strength in the model. Popular methods include LASSO, Ridge and elastic nets in regression, and Tree-based methods in Decision Trees, Random Forest, XGBoost, etc.

### 4.3. Phase 3: SPHM Modeling and Evaluation

As mentioned previously, this paper proposes the framework for SPHM and describes its first two phases in detail. In this phase, we provide a brief overview of the methods that can be used in modeling, evaluation and the deployment of the framework.

The selection of modeling methods is based on the manufacturing operation that is being considered. The most popular modeling approaches in the last decade have been data-driven, with some novel hybrid models being developed as well. Supervised learning methods, unsupervised methods as well as DL have come to the forefront in CBM and in the prediction of RUL. As we have presented in Section 2.5, there are a multitude of options to choose from for PHM modeling. Modeling methods not only include regression-based prediction, but also classification models for failure detection that determine whether a component will fail or not.

Evaluation metrics for models can be chosen based on the type of data-driven or hybrid models. There are metrics such as RMSE, Mean Absolute Error (MAE), coefficient of determination (R-Squared), adjusted R-squared and Mallow’s CP used in evaluation and assessment of regression models. Classification models are generally evaluated using a confusion matrix, in which the True Positive, True Negative, False Positive and False Negative classifications are recorded. Based on the confusion matrix, metrics such as accuracy, precision, sensitivity, specificity, F1 score, etc., are calculated and used in evaluating a classifier. Deep Learning methods can also be evaluated based on their predictive power by using accuracy as an evaluation metric.

Deploying SPHM models in the field requires development of apps, either web-based or mobile, so that engineers and technicians can analyze and assess the current state of operations based on real-time data. Digital-Twin-driven modeling consists of a digital replica of the shopfloor setup that is synced in real-time. A review of the development of such applications and their deployment is being considered a separate research area and is proposed as future work.

## 5. Case Study: Milling Machine Operation

Milling is one of the fundamental operations in manufacturing engineering and is essential in most, if not all shopfloors. It is an ideal starting point to analyze the SPHM framework in manufacturing and is useful in providing a basis to understanding how even a simple operation can generate such complex data. A typical milling machine setup comprises of the following components: spindle, cutting tool, base, workpiece, *X*-axis and *Y*-axis traversing mechanism, and a table upon which the workpieces are mounted. The cutting tool is used to remove material from the workpiece by moving it along the different axes via the machine table motion. Old milling machines are operated manually by using the mechanisms to move the cutting tool, whereas newer machines with Computer Numerical Control (CNC) controllers are equipped with a wide range of sensors and automated tool changing mechanisms depending upon the specifics of the operation. The experimental data considered for this paper were developed by Berkeley University, California [79,80].

In this use-case, we apply the first two phases of the proposed SPHM framework, and aim at understanding the setup, cleaning and preprocessing of the data, preprocessing the signals, extracting relevant features and selecting the final set of features.

### 5.1. Phase 1: Milling Machine Setup and Data Acquisition

#### 5.1.1. Milling Machine and Sensor Setup

**Figure 3:**
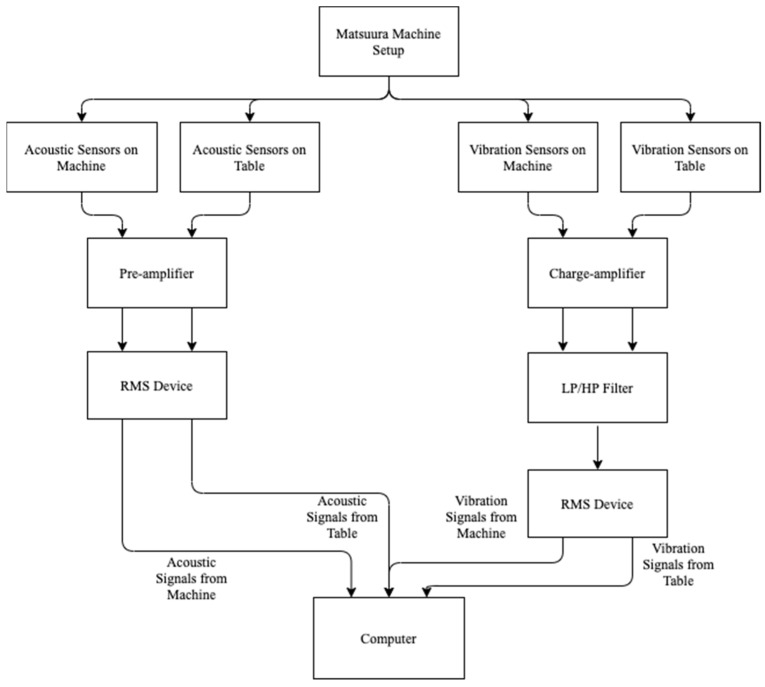
Milling operation setup, adapted from [79].

The setup used consists of an MC-510V Matsuura machine along with a table that it is mounted on. There are three sets of sensors: acoustic emission sensors, vibration sensors and current sensors. The acoustic emission sensor is the WD 925 model by the Physical Acoustic Group that has a frequency range of up to 2 MHz. This sensor is secured to a clamping support. The acoustic emission signals are passed through a model 1801 preamplifier built by Dunegan/Endevco. The preamplifier has an in-built 50 kHz high-pass filter. Further amplification of the signals is performed by using a DE model 203 A (Dunegan/Endevco). These signals then pass through a custom-made RMS meter with the time constant set to 8.0 ms. Then, these signals are fed through a UMK-SE 11,25 cable made by Phoenix Contact that is linked to an MIO-16 board made by National Instruments for high-speed data acquisition. Another acoustic emission sensor is mounted on the spindle and the signals follow the same path via the preamplifier, filter, amplifier and RMS meter to the data acquisition board. The vibration sensor used is an accelerometer built by Endevco, Model 7201-50, that has a frequency range of up to 13 kHz. Vibration signals pass through a model 104 charge amplifier made by Endevco and then through an Itthaco 4302 Dual 24 dB octave filter (low-pass and high-pass). These signals then pass through a custom-made RMS meter and to the MIO-16 board using a UMK-SE 11,25 cable. The vibration sensors are mounted on both the table and the milling machine’s spindle. An OMRON K3TB-A1015 current converter feeds signals from one spindle motor current phase to the high-speed data acquisition board. Another current sensor, model CTA 213, built by Flexcore Division of Marlan and Associates, Inc., also feeds signals into the data acquisition board. See Figure 3 for the experimental setup showing the connections of acoustic and vibration sensors.

The selection of parameters for the experiment was chosen based on manufacturers’ standards and industry specifications. Two types of inserts were selected for the cutting tool, KC710 and K420. They are resistant to wear and can function in environments that involve high friction. The materials for the workpieces were stainless-steel J45 and cast iron. Other important parameters included the setting of the speed of the cutting tool to 200 m/min, Depth of Cut (DOC) of the two settings of 1.5 and 0.5 mm and feeds to two settings of 413 and 206.5 mm/min. The combinations of the numbers of parameters resulted in 8 different settings under which the milling machine could operate.

The experimental data from [79,80] consisted of 16 cases with varying DOC, feed and materials. These 16 cases were used as experimental conditions and run multiple times. The numbers of runs were determined by evaluating flank wear on the cutting face of the tool, by taking measurements at intermittent but non-uniform intervals.

#### 5.1.2. Data Collection and Understanding

The data were recorded as a ‘struct array’ using MATLAB [81] software. The dataset consists of 13 features, out of which 6 are derived from sensor readings. The description of the dataset can be found in Table 4. There are 16 cases, with the DOC, feed and material kept constant for each case. Each case consists of a varying number of runs that are dependent on VB, the degree of flank wear. VB measurements were recorded at irregular intervals up to the limit when significant wear was observed. If we look closely at the MATLAB file, we notice that each of the values under the sensor features (smcAC, smcDC, vib_table, vib_spindle, AE_table and AE_spindle) comprise of a 9000 × 1 dimensional vector. This is because the sensor’s signals are amplified and filtered before being captured, resulting in measurements that are of high dimensions. A view of the first few rows of the dataset can be seen in Table A1 of Appendix A, showing the highly dimensional values in sensor readings. These features need to be preprocessed and transformed so that they can be analyzed more thoroughly. We also note some missing values, and possibly some outliers that can be troublesome while conducting a data-driven approach. This dataset requires preparation and preprocessing for it to be suitable for use in modeling.

### 5.2. Phase 2: Data Preparation and Analysis

#### 5.2.1. Data Cleaning and Preprocessing

**Figure 4:**
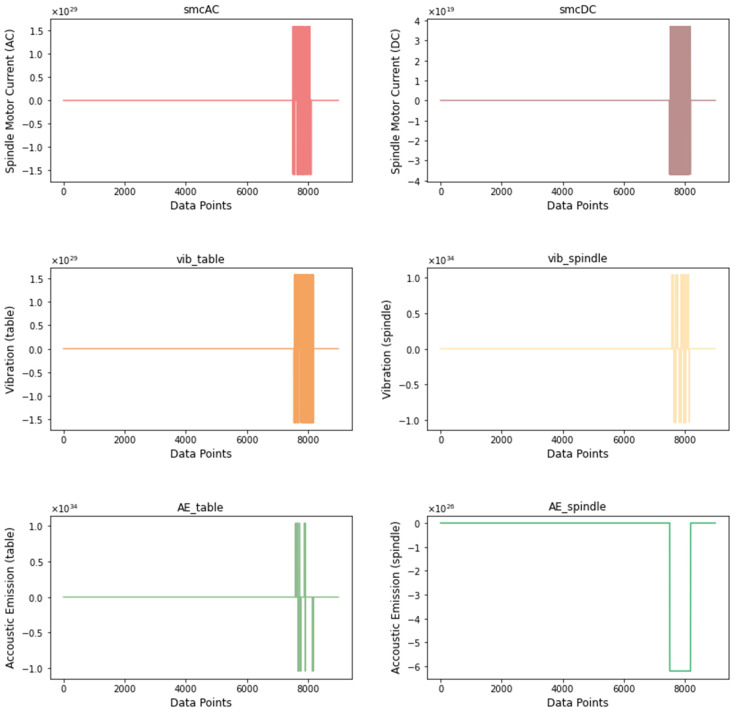
Signatures from the six sensors showing an outlier for case 2, run 1.

**Figure A1:**
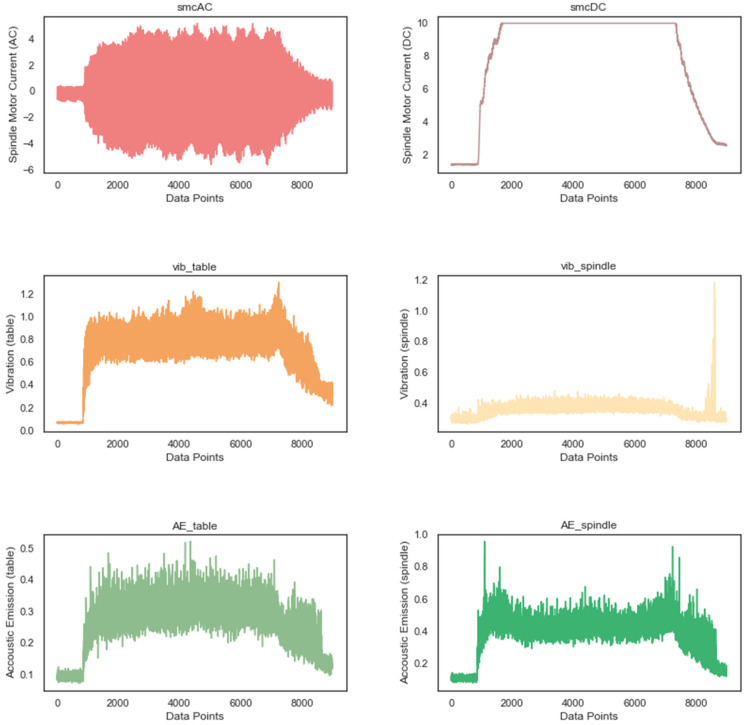
Signatures from the six sensor signals for case 1, run 11.

VB is the most important feature in this dataset since RUL assessment and condition monitoring are performed based on VB values. If we observe the dataset closely, we notice that there are missing values in the VB column. This is because VB measurements were taken are irregular intervals until the degradation limit. There were 21 instances identified that contained missing VB values. Since VB is crucial to any analysis that we wish to conduct, the appropriate strategy in this case is to delete the instances in which missing values are observed. After removing the instances with the missing VB values, the dataset is reduced to 146 instances. The next step in this process is to identify any outliers in the data. Figure 4 shows the signal signatures from the six sensors for run 1 of case 2. Compared to signals from other instances (see Figure A1 in Appendix A), the ones from Run 1 of Case 2 are of a much higher magnitude. The sensor measurements for this case have peaks at the following magnitudes: smcAC at 10^29^, smcDC at 10^19^, vib_table at 10^29^, vib_spindle at 10^34^, AE_table at 10^34^ and AE_spindle at 10^26^. The rectangular-shaped peaks observed for this case cannot be attributed to any filtering or operation from the experiment. After comparing this anomalous instance against other instances, we can confirm that it is an outlier that is most likely due to measurement error. Similarly, all the instances in the dataset are scanned for abnormalities. We observe that there is only one instance of an outlier, and we choose to discard it under the circumstances, with the final dataset consisting of 145 instances. Before we move on to the next step, it is crucial to note that the data have not been scaled or normalized. This is because scaling/normalization is performed after all the features have been generated.

#### 5.2.2. Signal Preprocessing

The preprocessing of signals from sensors was conducted during the experimental setup, by Goebel et al. [79,80]. The signals from acoustic and vibration sensors were amplified in the range of ±5 V, according to the equipment threshold. The vib_table and vib_spindle signals were routed through a low-pass and high-pass filter, attenuating any frequency that did not meet the cut-off. The acoustic signals were fed through a high-pass filter to filter out any unwanted frequencies. Cut-off frequencies were identified based on graphical displays on an oscilloscope, with cut-offs of 400 Hz and 1 kHz set for the low-pass and high-pass filters, respectively. An equipment threshold of 8 kHz was set for the acoustic emission sensor, meaning that any frequency observed above that would not be due to machining operations and is filtered out. An RMS meter allowed the signals to undergo some additional preprocessing by smoothing them.

#### 5.2.3. Feature Extraction

In this step, feature extraction methods were applied to generate features in the time domain and frequency domain. The methods applied in feature extraction are ones that have been proven to be suitable for machining operations. Time-domain features were extracted using the prescribed feature set in Table 2. This method generates 54 features, i.e., 9 new features for each of the 6 signals. Frequency-domain features were generated using the prescribed feature set in Table 3, generating an additional 42 features, i.e., 7 new features for each of the 6 input signals. The total generated features are 96, which is a highly dimensional feature set.

Once the features are extracted, we note that some of the new features are on varying scales that could skew the modeling approach. Features based on Kurtosis of Band Power and Relative Spectral Peak per Band consist of values that are significantly higher than values of features based on Mean of Band Power and Variance of Band Power. Therefore, we choose to apply min–max normalization, a method shown in Equation (1). This ensures that all the features are on the same normalized scale.

#### 5.2.4. Feature Evaluation and Selection

**Figure A2:**
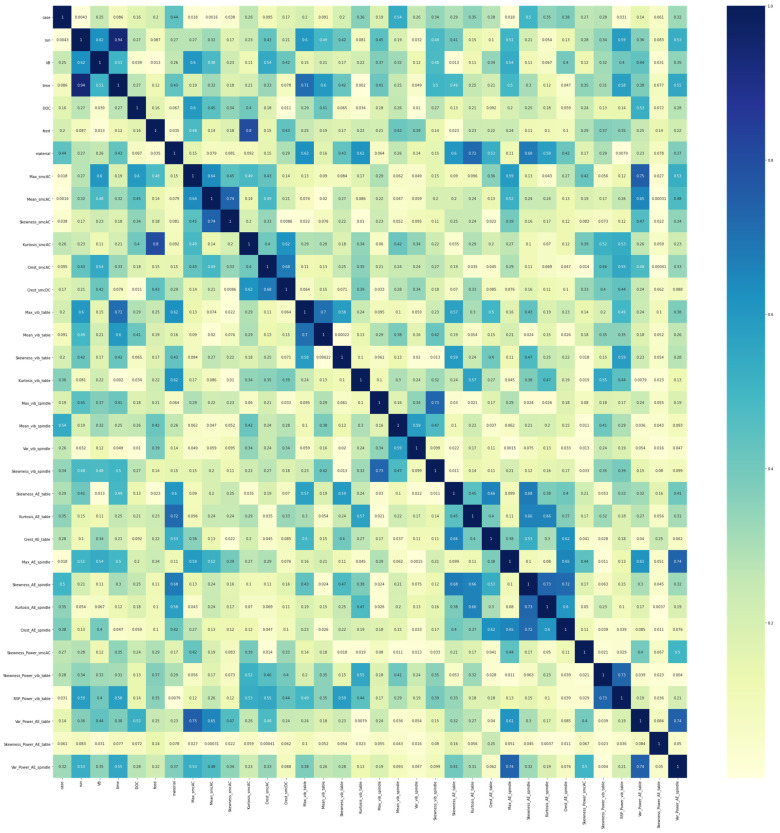
Correlation matrix of final set of features.

The feature set after extracting features from signals consists of a total of 103 features. Seven features are parameters of the experiment: case, run, VB, time, DOC, feed and material. The other 96 features are extracted as discussed in the previous step. The data in their current state consist of 145 instances and 103 features. To ensure that the curse of dimensionality is avoided in the modeling phase, the most important features to predict VB and the RUL need to be identified. As a first step, we perform a univariate analysis by calculating the correlation coefficients between the individual features and the response variable VB. All 96 newly generated features are considered, ignoring the 7 experimental parameters since they are important and must be included in the analysis. The correlation coefficients are then ranked in descending order, allowing us to observe which of the newly generated features have a relationship with the response. Next, we calculate the correlation matrix for all 96 features and use the univariate ranking with VB to choose the best features. Feature pairs that have a Pearson correlation coefficient of 0.75 or more are considered for potential elimination by comparing their relationship with VB. From the feature pair, the one that has a higher correlation coefficient with VB is retained, while the other is dropped. This method allows us to identify the most important features without having to specify the number of features, which is a big research problem by itself. The final dataset consists of 34 features, out of which 27 features are extracted from the input signals, 6 of them are experimental parameters, and 1 is the response variable. The correlation matrix for the final set of features is shown in Figure A2 of Appendix A. As we can see, features that have a pairwise correlation coefficient of 0.75 or more are noted. The ranked correlation coefficients of these features with VB are compared, and features are removed accordingly. This is a simple, yet effective strategy to eliminate features that have no strength in the analysis.

## 6. Results

The application of Phases 1 and 2 of the proposed SPHM framework to milling machine operations provides us with a thoroughly prepared dataset for ML and DL purposes. The milling machine experimental setup is reviewed, and the operating parameters are noted. Details on the acquisition of the data are listed along with a data dictionary consisting of the attributes and their description. The final dataset is cleaned of missing values and outliers. Signals are preprocessed, and suitable features are extracted based on prior knowledge and proven extraction methods. The final set of features is selected based on a comparison of pairwise correlation coefficients and the ranking of the correlation coefficients with the response variable, VB. The link between the steps in the first two phases of SPHM and its application to the milling machine case is shown in Table 5. The SPHM framework thus far has proven to be an effective methodology to setup the experiment, acquire data, prepare data, preprocess data and the signals, extract features and evaluate and select features.

## 7. Conclusions and Future Work

This paper provided an in-depth understanding of PHM and maintenance approaches to manufacturing. The different approaches and challenges to PHM were outlined, and the current trends in PHM research were reviewed. A unique SPHM framework that is interoperable to all areas of manufacturing was proposed and the multifaceted SPHM framework was described in 3 phases—Phase 1: Setup and Data Acquisition, Phase 2: Data Preparation and Analysis and Phase 3: SPHM Modeling and Evaluation. In this paper, Phase 1 and Phase 2 were discussed in detail, with the nuances and the elaboration of Phase 3 considered as future work. In future studies, we wish to focus on advanced Deep Learning methods as a part of SPHM Phase 3 and compare their performance with baseline Machine Learning methods, such as regression, SVM, etc.

## Figures and Tables

**Table 1:** Advantages and disadvantages of prognostics modeling approaches.

Modeling Approach	Advantages	Disadvantages
***Physics-based models***	No-randomness involved, resulting in accurate analysis Can be used with small datasets	Complexity in implementing and require intricate laboratory settings Expertise in system modeling is required
***Data-driven models***	Little expertise in system modeling is required Easy implementation Cost-effective since there is no need to simulate operating conditions	Lack of suitable data Low quality of available data Difficulty in attributing causes of failure
***Hybrid models***	Can be used with small datasets Not that difficult to implement Flexibility in modeling	Selection of parameters involves high level of complexity Balanced data with failure events required

**Table 2:** Time-domain features for commonly observed sensor signals for machining according to [72].

Index	Feature	Description
1	Maximum	$X_{MAX}=Max\left(x_{i}\right)$
2	Mean	$\mu =\frac{1}{n}\;\overset{n}{\underset{i=1}{\sum }}x_{i}$
3	Root Mean Square	$X_{RMS}=\sqrt{\frac{1}{n}\;\overset{n}{\underset{i=1}{\sum }}x_{i}^{2}}$
4	Variance	$X_{V}=\frac{\sum_{i=1}^{n}{\left(x_{i}-\mu \right)}^{2}}{n-1}$
5	Standard Deviation	$\sigma =\sqrt{\frac{\sum_{i=1}^{n}{\left(x_{i}-\mu \right)}^{2}}{n-1}}$
6	Skewness	$X_{V}=\frac{1}{n}\;\frac{\sum_{i=1}^{n}{\left(x_{i}-\mu \right)}^{3}}{\sigma^{3}}$
7	Kurtosis	$X_{V}=\frac{1}{n}\;\frac{\sum_{i=1}^{n}{\left(x_{i}-\mu \right)}^{4}}{\sigma^{4}}$
8	Peak-to-Peak	$X_{P2P}=max\left(x_{i}\right)-min\left(x_{i}\right)$
9	Crest Factor	$X_{CF}=\frac{max\left(x_{i}\right)}{\sqrt{\frac{1}{n}\;\sum_{i=1}^{n}x_{i}{}^{2}\;}}$

**Table 3:** Frequency-domain features for commonly observed sensor signals for machining according to [72].

Index	Feature	Description
1	Maximum Band Power Spectrum	$S_{MAX}=Max\left(S{\left(f\right)}_{i}\right)$
2	Sum of Band Power Spectrum	$S_{SBP}=\overset{n}{\underset{i=1}{\sum }}S{\left(f\right)}_{i}$
3	Mean of Band Power Spectrum	$S_{\mu }=\frac{1}{n}\;\;\overset{n}{\underset{i=1}{\sum }}S{\left(f\right)}_{i}$
4	Variance of Band Power Spectrum	$S_{V}=\frac{\sum_{i=1}^{n}{\left(S{\left(f\right)}_{i}-S_{\mu }\right)}^{2}}{n-1}$
5	Skewness of Band Power Spectrum	$S_{S}=\frac{1}{n}\;\frac{\sum_{i=1}^{n}{\left(S{\left(f\right)}_{i}-S_{\mu }\right)}^{3}}{S_{V}{}^{\frac{3}{2}}}$
6	Kurtosis of Band Power Spectrum	$S_{S}=\frac{1}{n}\;\frac{\sum_{i=1}^{n}{\left(S{\left(f\right)}_{i}-S_{\mu }\right)}^{4}}{S_{V}{}^{\frac{4}{2}}}$
7	Relative Spectral Peak per Band	$X_{CF}=\frac{max\left(S{\left(f\right)}_{i}\right)}{\sqrt{\frac{1}{n}\;\sum_{i=1}^{n}S{\left(f\right)}_{i}\;}}$

**Table 4:** Features of the milling dataset and their description.

Feature Name	Feature Description
case	Cases from number 1 to 16
run	Counting the runs in each case
VB	Flank wear observed in the cutting tool, not observed after each run
time	Time taken for each experiment, resets after completion of each case
DOC	Depth of Cut, kept constant in each case
feed	Feed, kept constant in each case
material	Material, kept constant in each case
smcAC	AC current at spindle motor
smcDC	DC current at spindle motor
vib_table	Vibration measured at table
vib_spindle	Vibration measured at spindle
AE_table	Acoustic emission measured at table
AE_spindle	Acoustic emission measured at spindle

**Table 5:** SPHM Phases implemented on milling data.

SPHM Phase	Steps	Relevant Section	Implementation on Use-Case
Phase 1: Setup and Data Acquisition	Shopfloor Setup	Section 5.1.1	Milling operation setup and sensors used reviewed
	Data Collection and Understanding	Section 5.1.2	Dataset explored, features described and preliminary investigation of data conducted
Phase 2: Data Preparation and Analysis	Data Cleaning and Preprocessing	Section 5.2.1	Missing values identified and eliminated Outliers visualized and removed
		Section 5.2.3	Feature Scaling
	Signal Processing	Section 5.2.2	Signal Preprocessing steps: Amplification, filtering, RMS
	Feature Extraction	Section 5.2.3	Features extracted in time domain and frequency domain
	Feature Evaluation and Selection	Section 5.2.4	Correlation-based feature selection

## Data Availability

The data used in this article are publicly available at https://ti.arc.nasa.gov/tech/dash/groups/pcoe/prognostic-data-repository/, Accessed on: 20 July 2021.

## Appendix A

**Table A1:** Snapshot of the dataset.

Case	Run	VB	Time	DOC	Feed	Material	smcAC	smcDC	vib_table	vib_spindle	AE_table	AE_spindle
1	1	0	2	1.5	0.5	1	9000 × 1 dim	9000 × 1 dim	9000 × 1 dim	9000 × 1 dim	9000 × 1 dim	9000 × 1 dim
1	2	NaN	4	1.5	0.5	1	9000 × 1 dim	9000 × 1 dim	9000 × 1 dim	9000 × 1 dim	9000 × 1 dim	9000 × 1 dim
1	3	NaN	6	1.5	0.5	1	9000 × 1 dim	9000 × 1 dim	9000 × 1 dim	9000 × 1 dim	9000 × 1 dim	9000 × 1 dim
1	4	0.11	7	1.5	0.5	1	9000 × 1 dim	9000 × 1 dim	9000 × 1 dim	9000 × 1 dim	9000 × 1 dim	9000 × 1 dim
1	5	NaN	11	1.5	0.5	1	9000 × 1 dim	9000 × 1 dim	9000 × 1 dim	9000 × 1 dim	9000 × 1 dim	9000 × 1 dim
